# Spontaneous Bladder Rupture After Binge Drinking

**DOI:** 10.7759/cureus.48107

**Published:** 2023-11-01

**Authors:** Jeremy Miller, Andrew McCague

**Affiliations:** 1 Surgery, Desert Regional Medical Center, Palm Springs, USA; 2 Trauma and Acute Care Surgery, Desert Regional Medical Center, Palm Springs, USA

**Keywords:** spontaneous bladder rupture, bladder repair, surgery, alcohol, bladder rupture

## Abstract

Spontaneous bladder rupture is a rare cause of the acute abdomen. Alcohol has been described as one of the most common causes of spontaneous bladder rupture. We present the case of a 42-year-old male who presented to our Level I Trauma Center complaining of abdominal pain and difficulty urinating after an evening of drinking. Initial workup revealed free air and fluid within the abdomen and a Foley catheter within the peritoneal cavity. He was taken to the operating room emergently for exploration and was found to have a bladder rupture that was repaired. Post-operatively he recovered without complication. The often missed or delayed diagnosis of spontaneous bladder ruptures can increase morbidity and mortality. It is important to keep spontaneous bladder rupture in the differential when evaluating a patient with abdominal pain.

## Introduction

Trauma is the most common cause of bladder rupture overall. Rarely, spontaneous bladder rupture can occur. Patients who present with spontaneous bladder rupture often provide a limited history and vague complaints of abdominal pain and urinary retention. Alcohol has been reported as one of the most common factors related to spontaneous bladder rupture. Here we present a rare case of a patient found to have a spontaneous bladder rupture after an evening of heavy drinking.

## Case presentation

Here we present a unique case of a 42-year-old male who presented to the Emergency Department (ED) of our Level I Trauma Center complaining of abdominal pain and inability to urinate. His pain began the evening prior after a period of heavy drinking. He says he went to sleep but woke up with pain. He attempted to urinate and noticed blood in his urine. He denied any history of trauma. He presented to the ED for evaluation.

The patient has a past medical history of hypertension and a surgical history of a left Achilles tendon repair. Specifically, he denied any history of previous bladder trauma, instrumentation, diverticular or masses. He denied any previous hematuria. He is a nonsmoker, drinks daily, and denies drug use. He denied a family history of diabetes or cancer.

**Figure 1:**
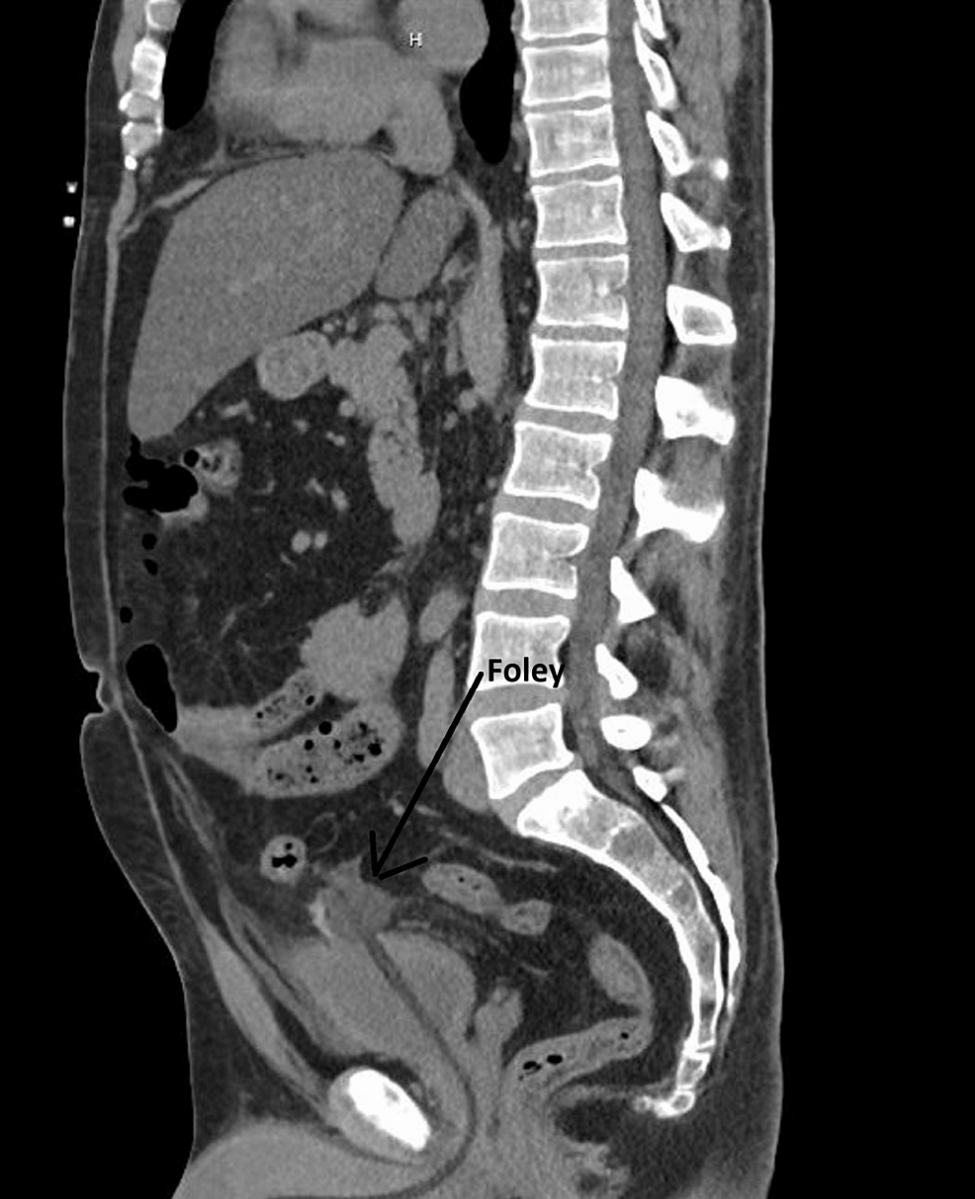
Computerized tomography of the abdomen and pelvis showing small free air, free fluid, and a Foley catheter protruding through the ruptured bladder

The patient’s chief complaint upon presentation to the ED was urinary retention. A Foley was placed and two liters of blood-tinged urine returned. Initial laboratory results are presented in Table 1. He underwent a computerized tomography scan of his abdomen and pelvis, which reported: "Pneumoperitoneum, compatible with bowel perforation; the Foley catheter has perforated through the bladder dome, with possible extravasation of urine" (Figure 1). An emergent surgery consult was obtained and the patient was taken to the operating room emergently for exploration.

**Table 1:** Laboratory results on initial presentation

Laboratory value	Patient result	Normal reference range
White blood cell	10.6 x 10^3^/mm^3^	4.5-11 x 10^3^/mm^3^
Hemoglobin	15.4 g/dL	13-18 d/dL
Hematocrit	44.1%	37-49%
Platelets	191 x 10^3^/µL	130-400 x 10^3^/µL
Sodium	135 mEq/L	135-145 mEq/L
Potassium	5.2 mEq/L	3.4-5.0 mEq/L
Chloride	102 mEq/L	95-108 mEq/L
Carbon dioxide	21.0 mEq/L	20-32 mEq/L
Blood urine nitrogen	23 mg/dL	8-25 mg/dL
Creatinine	2.4 mg/dL	95-108 mg/dL
Blood glucose	115 mg/dL	70-110 mg/dL

Intraoperatively, the patient underwent an exploratory laparotomy where a large, 10 cm, defect was found at the dome of the bladder. The bladder injury was repaired in layers using a chromic suture. Postoperatively he recovered and a Foley was kept in place on discharge. He followed up in the clinic two weeks post-trauma and the Foley catheter was removed after a normal cystogram. He recovered well post-discharge and has not required readmission.

## Discussion

Spontaneous bladder rupture is a rare but potentially life-threatening condition that has been reported to occur in one of every 126,000 people [1,2]. Given its often-delayed presentation, the diagnosis can be difficult and misdiagnoses contribute to its potentially high mortality rate [3].

Overall, bladder rupture is most commonly attributed to trauma. Over 96% of cases have a traumatic origin [4]. High-speed blunt trauma or penetrating injuries to the lower abdomen can cause a rupture at the dome of the bladder with leakage of urine into the abdominal cavity [5]. The cause of spontaneous bladder rupture is not always obvious. A paper published by Zhang et al. in 2021 presented an extensive literature review where 713 published spontaneous bladder rupture patients were analyzed [3]. In their paper, they report that the most common cause was alcohol (39.27%) followed by urinary tract obstruction (18.37%), bladder tumors or inflammation (12.76%), pregnancy (7.57%), bladder dysfunction (5.89%), previous radiation to the pelvis (3.51%), and previous bladder surgery or history of bladder diverticulum (3.37%), neurological or psychiatric diseases (1.4%), strenuous exercise (0.56%), pelvic disease invasion (0.42%), long term hemodialysis (0.28%), and idiopathic causes (6.95%) [3].

The pathophysiology of spontaneous bladder rupture includes the combination of bladder overdistention along bladder wall thinning [5]. Causes of overdistention may include urinary retention in benign prostatic hyperplasia, urethral strictures, pelvic organ prolapse, inflammation, and malignancy [5]. Bladder wall weakness is attributed to previous bladder procedures including endoscopic urologic treatments such as catheterization, cystoscopy, or more invasive procedures such as transurethral resection of bladder tumor or cystectomy [2]. Other causes of bladder wall weakening can be seen with chronic distention, malignancies, post-menopausal women, and bladder diverticulum [2]. A bladder diverticulum is defined as a pouch protruding from a bladder wall; these can be either congenital or acquired. Acquired diverticula are seen more often, usually secondary to long-term outflow obstruction or neurogenic bladder [4].

As reported by Zhang et al., alcohol is considered the most common predisposing factor for spontaneous bladder ruptures [3]. Heavy alcohol consumption has a diuretic impact that causes the bladder to fill quickly with a rapid rise in intraluminal bladder pressure [1,2]. Alcohol also leads to paralysis of the central and peripheral nervous system and bladder sensorium dysfunction preventing the bladder's normal micturition response [2]. The frequent emesis seen in alcoholic patients has been reported to increase intraabdominal pressure that can transmit to a weakened bladder wall and lead to rupture [2]. Patients with a history of alcohol use give poor histories with lapses in memory of certain events and often frequent complaints of nonspecific abdominal pain [4]. Consequently, these cases are often complicated due to a delayed diagnosis and can lead to increased morbidity and mortality [6].

Bladder ruptures come in two general categories - extraperitoneal or intraperitoneal [2]. About 60-65% of bladder ruptures are extraperitoneal, while intraperitoneal make up about 25% [2]. The presentation can be different depending on the location of the rupture. Patients with extraperitoneal bladder rupture often present with pelvic pain, distended abdomen, difficulty urinating, oliguria/anuria, and fevers [1,3]. Intraperitoneal bladder ruptures are often less specific and are frequently accompanied by abdominal pain, macroscopic hematuria, difficulty urinating, or inability to urinate [2]. Intraperitoneal bladder ruptures are often associated with urinary ascites and carry an increased risk of infection, morbidity, and mortality [1].

The varied causes and presentations often lead to delayed or misdiagnoses in patients with spontaneous bladder ruptures. Common differential diagnoses include acute abdomen, inflammatory bowel disease, bladder tumors or inflammation, and renal failure [3]. Signs of ascites are common in these patients but there may be delays in hematuria suggesting a rupture. Red blood cells on urinalysis may not be visible for 24 to 36 hours after rupture [6]. The risk of mortality increased significantly after 24 to 48 hours of symptoms [6]. The possibility of bladder rupture should always be included in patients presenting with abdominal pain and difficulty urinating.

Most cases are diagnosed at the time of surgery for a presumed acute abdomen. Laboratory tests may look similar to those of renal failure with elevated serum creatinine, urine nitrogen, and potassium levels [1]. Retrograde or CT cystogram is the diagnostic modality of choice for patients with suspected spontaneous bladder rupture because of the high diagnostic accuracy and ability to evaluate structures of the abdomen and pelvis [1,6]. When ascites are present, paracentesis can be used to further characterize intraperitoneal bladder rupture when elevated creatinine levels are in the ascites fluid [6].

An early and accurate diagnosis followed by prompt surgical intervention is the key to a good outcome [3]. Surgical treatment is generally the first-line recommendation to treat spontaneous bladder ruptures [1]. When patient conditions permit, a laparoscopic approach is ideal given its flexibility and ability to explore the whole abdomen as well as perform a repair [1]. Zhang et al. reported in their 2021 literature review that 84.57% of patients were treated with an open repair and most were for intraperitoneal rupture [3]. They also reported that 1.12% of patients were treated with laparoscopic techniques and all were intraperitoneal repairs [3]. Nonoperative management has been reported for selected patients with indwelling catheters [1]. In general, extraperitoneal ruptures can be managed with a catheter alone while intraperitoneal ruptures require surgical repair and closure.

In our case presented above, this 42 year-old-male presented with abdominal pain and difficulty urinating after a night of heavy drinking. He was taken to the operating room for an acute abdomen where an isolated bladder injury was found and repaired. The patient denied any previous trauma. His documented alcohol use makes the history unreliable.

## Conclusions

We present a rare case of a spontaneous bladder rupture after an evening of heavy drinking. The uncommon nature, often-limited history, and vague abdominal complaints often lead to a delayed or missed diagnosis. The case we present above is that of a 42-year-old male who presented to the ED with abdominal pain, later found to be caused by a spontaneous bladder rupture. He was treated with open bladder repair and recovered without additional sequala. This case adds to the body of literature on this rare disease entity. Early diagnosis and treatment are vital to a good outcome.
